# Expression of Anion Exchanger 1 (AE1) and Its Potential Involvement in Human Granulosa Cell Physiology: An In Vitro Pilot Study

**DOI:** 10.3390/biom16071004

**Published:** 2026-07-09

**Authors:** Loris Marin, Chiara Sabbadin, Claudia Maria Radu, Paola Brun, Carolina Frison, Giuseppe Gullo, Decio Armanini, Luciana Bordin, Eugenio Ragazzi, Guido Ambrosini, Alessandra Andrisani

**Affiliations:** 1 Department of Women’s and Children’s Health, University of Padova, 35128 Padova, Italy; loris.marin@unipd.it (L.M.); guido.ambrosini@unipd.it (G.A.); alessandra.andrisani@unipd.it (A.A.); 2 Endocrine Unit, Department of Medicine DIMED, University of Padova, 35128 Padova, Italy; chiara.sabbadin@unipd.it (C.S.); decio.armanini@unipd.it (D.A.); 3 Thrombotic and Haemorrhagic Diseases Unit, Department of Medicine-DIMED, University of Padova, 35128 Padova, Italy; claudiamaria.radu@unipd.it; 4 Department of Molecular Medicine DMM, University of Padova, 35128 Padova, Italy; paola.brun.1@unipd.it (P.B.); carolina.frison@studenti.unipd.it (C.F.); 5 Department of Obstetrics and Gynecology, Villa Sofia Cervello Hospital, University of Palermo, 90146 Palermo, Italy; gullogiuseppe@libero.it; 6 Studium Patavinum, University of Padova, 35122 Padova, Italy; eugenio.ragazzi@unipd.it

**Keywords:** granulosa cells, anion exchanger 1 (AE1), endometriosis, follicular fluid (FF), 4,4′-diisothiocyanatostilbene-2,2′-disulfonic acid (DIDS)

## Abstract

The anion exchanger 1 (AE1), traditionally known for its role in erythrocyte anion transport and acid–base homeostasis, has recently been identified in non-erythroid tissues, suggesting broader physiological functions. In the present study, we investigated for the first time the expression and potential role of AE1 in human ovarian granulosa cells (GCs) obtained from women with endometriosis (ENDO-GCs) or male factor infertility controls (MF-GCs). Cells were cultured in the presence of follicular fluid derived from control (FF-MF) or endometriosis patients (FF-ENDO), and DIDS-sensitive anion exchange activity was pharmacologically inhibited using 4,4′-diisothiocyanatostilbene-2,2′-disulfonic acid (DIDS). AE1 expression was evaluated at both protein and transcript levels together with markers of proliferation, inflammation, and steroidogenesis. The results demonstrate that AE1 is constitutively expressed in GCs and may contribute to granulosa cell homeostasis. Inhibition of DIDS-sensitive anion exchange activity inhibited cell proliferation, shifted cell morphology toward a fibroblast-like phenotype, and reduced estradiol and progesterone secretion and inflammatory (IL-6) gene transcription. Notably, MF-GCs cultured in FF-MF exhibited compensatory upregulation of AE1, whereas ENDO-GCs and cells exposed to FF-ENDO showed impaired adaptive responses and generalized transcriptional suppression. These findings provide preliminary evidence supporting a role for DIDS-sensitive anion exchange activity in granulosa cell physiology and warrant further studies to clarify the specific contribution of AE1 to follicular dysfunction associated with endometriosis. However, the specific mechanistic contribution of AE1 could not be established and will require further functional and genetic investigations.

## 1. Introduction

Granulosa cells (GCs) are specialized somatic cells that surround the oocyte within the ovarian follicle and play a pivotal role in folliculogenesis, oocyte maturation, and endocrine regulation. They form a metabolically active and hormonally responsive compartment that supports oocyte growth through bidirectional communication mediated by gap junctions, paracrine factors, and extracellular vesicles [1]. The functional integrity of granulosa cells is essential for normal ovarian physiology, as they are the primary source of estradiol and other steroid hormones that regulate follicular development and reproductive cyclicity [2].

The survival, proliferation, and differentiation of granulosa cells are tightly regulated by gonadotropins—particularly follicle-stimulating hormone (FSH)—and by local intra-follicular factors such as growth differentiation factor 9 (GDF9), bone morphogenetic protein 15 (BMP15), and insulin-like growth factors (IGFs) [3]. In vitro studies have demonstrated that granulosa cells can maintain steroidogenic activity and responsiveness to FSH when cultured under appropriate conditions, although their survival is highly dependent on the composition of the culture medium and the presence of follicular fluid (FF) components [4,5].

Follicular fluid is a complex biological medium derived from both plasma transudation and secretions of granulosa and theca cells [6,7]. It contains a wide array of bioactive molecules—including hormones, cytokines, metabolites, and ions—that create a microenvironment essential for oocyte competence and follicular growth [8,9,10,11]. Recent evidence indicates that follicular fluid exerts direct regulatory effects on granulosa cell physiology. For example, exposure of cultured granulosa cells to autologous follicular fluid enhances estradiol production and aromatase (CYP19A1) expression, while simultaneously reducing apoptosis through activation of the PI3K/AKT and ERK1/2 signaling pathways [7]. These findings suggest that follicular fluid not only reflects the metabolic and hormonal state of the follicle but also acts as an active modulator of granulosa cell function [4].

Despite these advances, the mechanisms underlying the bidirectional relationship between granulosa cells and follicular fluid remain incompletely understood. While granulosa cells contribute to the composition of follicular fluid through the secretion of proteins, ions, and metabolites, their specific role in regulating the formation and dynamic remodeling of this fluid during folliculogenesis is still unclear [6]. Understanding this interplay is crucial, as alterations in follicular fluid composition have been associated with impaired oocyte quality, reduced fertilization rates, and ovarian dysfunction [6,7].

A growing body of evidence suggests that alterations in the follicular microenvironment represent a key mechanism linking ovarian dysfunction to endometriosis. Follicular fluid, which reflects the combined metabolic and secretory activity of granulosa and cumulus cells, is markedly altered in women with endometriosis, displaying increased oxidative stress, inflammatory mediators, and a profound metabolic shift toward glycolysis and lipid metabolism [12]. These changes are closely associated with impaired oocyte competence and reduced fertility outcomes. Importantly, FF is not merely a passive reservoir but an active signaling milieu capable of modulating both granulosa cell function and even promoting endometrial cell proliferation, thereby potentially contributing to disease progression [13].

Within this framework, ion transport and pH regulation may represent important yet underexplored determinants of follicular homeostasis. Recent evidence indicates that granulosa-derived cells can sense and respond to extracellular acidity and metabolic cues, highlighting the importance of ion transporters in adapting to the follicular microenvironment. Given that endometriosis is characterized by chronic inflammation, oxidative stress, and altered cellular metabolism—conditions known to disrupt intracellular pH and redox balance—anion exchangers may represent key regulators at the interface between granulosa cell physiology and FF composition [14].

Dysregulation of such mechanisms may amplify the acidic and oxidative conditions observed in endometriosis-associated follicles, thereby altering granulosa cell secretory activity and ultimately reshaping FF composition. In turn, this altered microenvironment may impair oocyte quality and reinforce the pathogenic loop linking follicular dysfunction to endometriosis. In this context, ion transport systems emerge as particularly intriguing regulators of granulosa cell function. Among them, the anion exchanger 1 (AE1), also known as Band 3 (encoded by *SLC4A1*), represents a compelling yet largely unexplored candidate. Well-characterized in erythrocytes and renal α-intercalated cells, where it mediates the electroneutral exchange of chloride (Cl^−^) and bicarbonate (HCO_3_^−^), thereby contributing to intracellular pH regulation, CO_2_ transport, osmotic balance, and cellular redox homeostasis [15], AE1 has increasingly been recognized for its broader roles in cellular homeostasis across different tissues [16].

Although direct evidence for AE1 involvement in granulosa cells is currently lacking, the established role of ion channels and transporters in endometriosis pathophysiology supports the plausibility of this hypothesis and highlights AE1 as a novel candidate deserving further investigation.

Transposing this knowledge to ovarian physiology opens new perspectives. If expressed in granulosa cells, AE1 could act as a key modulator of ionic gradients and acid–base balance, processes that are fundamental for intracellular pH sensing and regulation. Through these mechanisms, AE1 may influence granulosa cell survival, proliferation, and differentiation, as well as their secretory activity.

The aim of this study was to explore the presence and functional role of AE1 in granulosa cells in order to uncover a previously unrecognized layer of regulation in folliculogenesis. Such insights may help bridge the gap between cellular physiology and follicular fluid composition, ultimately improving our understanding of ovarian function and its dysregulation in pathological conditions.

## 2. Materials and Methods

This prospective, single-center study was conducted at the Assisted Reproductive Center of the University Hospital of Padua, Italy. Between September and December 2025, 32 infertile women scheduled for IVF treatment were assessed for eligibility; of these, 24 were enrolled, and written informed consent was obtained from all participants. The study was approved by the Institutional Review Board of the University Hospital of Padua (CET-ACEV 6150/AO/24) and conducted in accordance with the Declaration of Helsinki.

Exclusion criteria included polycystic ovary syndrome, diabetes, thyroid dysfunction, amenorrhea, active or chronic infections, autoimmune disorders, severe systemic diseases, and any history of malignant or premalignant gynecological pathology. Inclusion criteria required a confirmed diagnosis of endometriosis.

Participants were categorized into two groups: women whose partners had documented sperm abnormalities and were therefore classified as having male factor infertility (MF; *n* = 10), and women with endometriosis (ENDO; *n* = 14), confirmed surgically and/or by ultrasound imaging. Women with male factor infertility were selected as controls as they represent a population undergoing IVF for a cause unrelated to female reproductive pathology, with granulosa cells and follicular fluid presumed to reflect normal ovarian physiology. No formal power calculation was performed because this was an exploratory pilot study. The sample size (10 and 14 patients per group) was considered acceptable for preliminary investigation and hypothesis generation, in line with published recommendations for pilot studies [17].

### 2.1. Collection and Processing of Follicular Fluid (FF)

Follicular fluid (FF) was collected from women undergoing IVF treatment at the Assisted Reproductive Center. Controlled ovarian stimulation (COS) was performed using a short GnRH antagonist protocol with follitropin-alpha (Ovaleap^®^, Theramex, London, UK) and highly purified menotropin (Meriofert^®^, IBSA Institut Biochimique SA, Lugano, Switzerland), starting on day 2 or 3 of the menstrual cycle. The total gonadotropin dose and COS duration did not differ among patients. The FSH dose was individualized based on ovarian reserve parameters (antral follicle count and AMH levels). Daily subcutaneous ganirelix (0.25 mg; Orgalutran^®^, Organon, Amsterdam, The Netherlands) was administered when at least one follicle reached 14 mm in diameter and continued until human chorionic gonadotropin (hCG) administration. Ovulation triggering was performed with 250 µg recombinant hCG (Ovitrelle^®^, Merck Serono S.p.A., Darmstadt, Germany) when the leading follicle cohort exceeded 16 mm, followed 36 h later by oocyte retrieval. Follicles < 14 mm were not aspirated, in accordance with ESHRE guidelines [18].

During transvaginal oocyte retrieval, FF was collected from all punctured follicles. To minimize contamination with vaginal epithelial cells, the first aspiration tube from each patient was discarded. For each patient, FF samples from all follicles were pooled. The cellular content typically consisted of granulosa cells (GCs), both isolated and clustered, erythrocytes, and large epithelial cells.

Collected FF was centrifuged at 200× *g* for 10 min to separate GCs. The supernatant was then centrifuged at 4500× *g* for 10 min and stored at −20 °C. Upon thawing, FF underwent an additional centrifugation step (4500× *g*, 10 min), followed by sterile filtration (0.22 µm pore size) and storage at −20 °C until analysis [4].

To reduce variability in FF characteristics, samples from 2 to 3 women with the same infertility diagnosis (MF or ENDO, respectively) were pooled. Because the number of follicles recovered varied considerably among patients (Table S1), particularly between MF and ENDO groups, individual patients did not necessarily contribute equally to the pooled samples. Pooling was based on the amount of biological material recovered rather than on a fixed number of patients. Because MF patients generally yielded a higher number of follicles per retrieval cycle (typically 6–8 follicles), sufficient follicular fluid (FF) volumes and granulosa cell (GC) numbers were often obtained by pooling samples from two patients. In contrast, ENDO patients frequently produced fewer follicles (typically 3–4 follicles), requiring the pooling of samples from up to three patients to obtain comparable amounts of FF and GCs. Consequently, patients with a higher follicular yield occasionally contributed material to more than one independent pool, whereas samples from patients with lower follicular yields were combined with those from additional donors to generate sufficient biological material for each experiment.

To minimize potential intra-group bias, pooled samples were generated using material derived from patients of different ages and, within the ENDO group, from patients with varying disease stages. This strategy was adopted to reduce the influence of individual outliers and to obtain a biological milieu representative of each clinical condition. Each pool contained biological material exclusively from patients belonging to the same diagnostic group (either MF or ENDO), and no pool included material from both clinical groups.

Importantly, FF pooling and GC pooling were performed independently. Therefore, granulosa cells were not exposed to their corresponding individual FF samples, allowing the evaluation of disease-associated effects while minimizing the contribution of patient-specific follicular microenvironments. Using this approach, six independent pooled samples were generated for each experimental condition. It should be noted that, since patients with higher follicular yields occasionally contributed to more than one pool, complete statistical independence between all replicates cannot be guaranteed, and this represents an inherent limitation of the pooling approach.

### 2.2. GCs Preparation and Treatment

Isolated granulosa cells (GCs) were washed twice by centrifugation (200× *g*, 10 min, room temperature) using culture medium (CM) composed of Dulbecco’s Modified Eagle’s Medium/Ham’s F12 (DMEM/F12; Thermo Fisher Scientific, Waltham, MA, USA), supplemented with 8% fetal bovine serum (FBS), 10,000 U/mL penicillin, and 10,000 μg/mL streptomycin. Cells were then incubated at 37 °C in a humidified atmosphere with 5% CO_2_ for 24–36 h to allow viable GCs to adhere. Subsequently, cultures were rinsed with CM to eliminate non-adherent, apoptotic, and contaminating cells, and fresh medium was added.

After 7 days in culture, adherent cells were detached using TrypLE Express (Thermo Fisher Scientific) for 7 min at 37 °C, in accordance with the manufacturer’s instructions, and counted using a Makler chamber. Cells were seeded either in 12-well plates (5 × 10^4^ cells/well in 1 mL CM) or in T25 flasks (5 × 10^6^ cells in 5 mL CM), with or without supplementation of 20% FF derived from the different experimental groups. To reduce inter-individual variability, GCs obtained from 2 to 3 patients within the same group were pooled. Each experimental replicate therefore represents an independent biological pool derived from a distinct subset of patients, ensuring biological independence across the *n* = 6 replicates used in statistical analyses. Additional variability was minimized by including donors of different ages and, for endometriosis samples, different disease stages. Importantly, GC cultures were not exposed to their corresponding FF samples. Experimental conditions were maintained as previously described [5].

Cells were cultured at 37 °C in 5% CO_2_, and non-adherent elements were removed after 48 h. The medium was refreshed twice weekly. During the final 5 days of culture, medium replacement was discontinued; instead, the supernatant was collected at the end of incubation as conditioned culture medium (CCM), centrifuged, and stored for subsequent hormonal and cytokine analyses.

### 2.3. Replication Assay as Population Duplications (PDs) Count

The assay for the proliferation analysis was performed as previously described in [5]. Briefly, cells were enzymatically detached using TryplExpress (Gibco™, Thermo Fisher Scientific), resuspended with CM, and centrifuged at 750× *g* for 5 min at room temperature. The resulting pellet was resuspended in CM, and cell viability was assessed by trypan blue exclusion.

Cells (5 × 10^4^/well) were plated in 12-well plates in triplicate, cultured under the different FF conditions, and counted on days 7, 14, and 21. Population doubling (PD) values were calculated using the formula:

PD = (log NT − log N0)/log 2,

where NT represents the number of cells at harvest and N0 the initial number of seeded cells.

### 2.4. GCs Morphological Monitoring

GC morphology was monitored by acquiring phase-contrast images with a Nikon Eclipse TE2000S microscope (Nikon Corporation, Tokyo, Japan) coupled to a Cooper Surgical DC1 digital camera. Observations were carried out directly in the culture vessels, namely 12-well plates featuring a gridded bottom surface (Chemglass Life Sciences, Vineland, NJ, USA), allowing consistent field selection over time.

For each condition, a minimum of 200 cells distributed across 10 randomly chosen microscopic fields were recorded. Morphological features and phenotypic variations were quantified using CellProfiler 4.2.8 (https://cellprofiler.org/), an open-source software designed for automated image processing and high-content analysis. To enhance reproducibility, all image analyses were independently performed by two trained investigators. The collected measurements were then pooled and processed for statistical evaluation.

### 2.5. GCs AE1 Expression by Flow Cytometry

Cells were plated in 12-well culture plates and maintained for 7 days at 37 °C in the different experimental media. At the end of the incubation period, cells were harvested, transferred into centrifuge tubes, and washed three times with phosphate-buffered saline (PBS). Intracellular AE1 staining was carried out using the FIX & PERM kit (Invitrogen, Thermo Fisher Scientific, Waltham, MA, USA), following the manufacturer’s protocol. Briefly, GCs were incubated for 45 min at room temperature with mouse monoclonal anti-human AE1 antibody (MO 63103, Sigma-Aldrich, St. Louis, MO, USA), followed by incubation with a goat anti-mouse IgG (H+L) Alexa Fluor 488-conjugated secondary antibody (Invitrogen, Thermo Fisher Scientific, Waltham, MA, USA).

Cells were first gated based on forward scatter area (FSC-A) versus side scatter area (SSC-A) to exclude debris and identify the cell population of interest. Doublets were excluded by gating FSC-A versus FSC-W. fluorescence signals were acquired in the FITC channel, and the mean fluorescence intensity (MFI) was quantified. Samples stained with the secondary antibody alone were used as negative controls to assess background fluorescence. Data acquisition was performed using a CytoFLEX SRT flow cytometer (Beckman Coulter Life Sciences, Indianapolis, IN, USA), and data were analyzed using CytExpert 1.2.0 software (Beckman Coulter, Brea, CA, USA).

Quantitative evaluation of AE1 expression was obtained by calculating the difference between the mean fluorescence intensity (MFI) of stained samples and the corresponding autofluorescence controls, consisting of cells incubated with secondary antibodies alone.

### 2.6. AE1 Localization by Immunocytochemistry (ICC)

GCs were cultured on sterile glass coverslips positioned in 12-well plates and maintained in the different experimental media for 7 days, until reaching approximately 60–70% confluence. Following incubation, coverslips were collected and washed three times with phosphate-buffered saline (PBS). Cells were then fixed and permeabilized using FIX & PERM™ Cell Permeabilization Reagents (Invitrogen, Thermo Fisher Scientific, Waltham, MA, USA) for 15 min at room temperature, following the manufacturer’s protocol. After fixation, samples were washed again with PBS and incubated for 30 min at room temperature in PBS containing 3% fetal bovine serum (FBS) to block non-specific binding sites. Subsequently, cells were incubated overnight at 4 °C with a mouse monoclonal anti-AE1 antibody (from Sigma-Aldrich, Saint Louis, MO, USA). After washing, samples were exposed for 30 min at room temperature to an Alexa Fluor™ 488-conjugated goat anti-mouse IgG (H+L) secondary antibody (Invitrogen, Thermo Fisher Scientific). Coverslips were then rinsed with PBS prior to image acquisition. Nuclear staining was performed using DRAQ5™ Fluorescent Probe (Thermo Fisher Scientific) for 25 min at room temperature.

Fluorescence images were collected using a DMI6000CS fluorescence microscope (Leica Microsystems, Milan, Italy) with a DFC365FX camera (Leica Microsystems), and analyzed by the LAS-AF software 3.1.1 (Leica Microsystems). 

### 2.7. Measurement of Interleukin-6 (IL-6) in FF and CCM

IL-6 concentrations in FF and CCM were measured using the MAGLUMI IL-6 chemiluminescence immunoassay (CLIA; Snibe Diagnostic, Shenzhen, China) on automated MAGLUMI analyzers, as previously described [5]. Briefly, this assay is based on a sandwich immunoassay principle that generates a chemiluminescent signal proportional to IL-6 levels.

For CCM samples, IL-6 values were normalized to both the initial cytokine content of the medium and cell proliferation. Specifically, IL-6 production was calculated by dividing the final IL-6 concentration measured at the end of the incubation period by the IL-6 concentration initially present in the medium, and then further adjusting this value by the population doubling (PD) of the corresponding granulosa cells. Results are reported as mean ± standard deviation (SD).

### 2.8. Quantification of Estradiol (E2) and Progesterone (P4) in FF and CCM

E2 and P4 concentrations in FF and CCM were measured using Elecsys Estradiol III and Elecsys Progesterone III assays (Roche Diagnostics, Basel, Switzerland) on cobas^®^ e series analyzers, as previously described [5]. These electrochemiluminescence immunoassays (ECLIA) are based on the sandwich principle and generate a signal proportional to the analyte concentration.

For CCM samples, hormone levels were normalized to both the initial hormone content of the medium and cell proliferation. Briefly, the concentration measured at the end of incubation was divided by the baseline concentration in the medium and adjusted according to the population doubling of granulosa cells. Results are expressed as mean ± standard deviation (SD).

### 2.9. Quantification of Follicle-Stimulating Hormone (FSH) and Luteinizing Hormone (LH) in FF

FSH and LH levels in follicular fluid were determined using Elecsys FSH and Elecsys LH assays (Roche Diagnostics, Basel, Switzerland) on cobas^®^ e series analyzers, as previously described [5]. These assays are based on electrochemiluminescence immunoassay (ECLIA) technology employing monoclonal antibodies and producing a signal proportional to hormone concentration.

### 2.10. Total RNA Quantification

Total RNA was extracted using the SV Total RNA Isolation System (Promega, Milan, Italy), following the manufacturer’s instructions. Residual genomic DNA was eliminated through on-column DNase I digestion. For quantitative reverse transcription PCR, 5 µg of total RNA were analyzed using the iTaq Universal One-Step RT-qPCR Kit (Bio-Rad, Milan, Italy), which includes iScript RNase H/MMLV reverse transcriptase, hot-start iTaq DNA polymerase, and SYBR^®^ Green dye. Primer pairs were designed with Primer3 software 4.1.0, and obtained from Merck (Milan, Italy); primer sequences are listed in Table 1. Amplification reactions were performed on a QuantStudio Real-Time PCR System (Thermo Fisher Scientific, Milan, Italy) using 40 amplification cycles and an annealing temperature of 60 °C. Relative gene expression levels were normalized against β-actin gene (*ACTB*) expression.

### 2.11. Statistical Analysis

Cell population composition data (ELGCs, FLGCs, MLGCs, CLGCs, and NLGCs, expressed as percentages) were analysed separately for MF-GCs and ENDO-GCs preparations using Linear Mixed-Effects Models (LMM). A separate LMM was fitted for each cell preparation, with treatment (DMSO vs. DIDS) and FF condition (FF-MF vs. FF-ENDO) as fixed effects, along with their interaction. Individual replicates (*n* = 6 pairs per condition) were included as a random intercept term to account for the paired experimental design. Fixed effects significance was assessed using Type III ANOVA with Satterthwaite’s approximation for degrees of freedom. Post hoc pairwise comparisons were performed using estimated marginal means (emmeans package) with Bonferroni correction, evaluating the effect of DIDS versus DMSO within each FF condition, and differences between FF-MF and FF-ENDO within each treatment. Additionally, paired Wilcoxon signed-rank tests and paired *t*-tests were applied for each FF condition × cell type combination, with False Discovery Rate correction (Benjamini–Hochberg method). Data are presented as box plots showing median and interquartile range. To directly compare MF-GCs and ENDO-GCs preparations, an additional LMM was fitted for each cell type including preparation (MF-GCs vs. ENDO-GCs), treatment (DMSO vs. DIDS), and FF condition (FF-MF vs. FF-ENDO) as fixed effects with all two-way and three-way interactions, and individual replicates as a random intercept. Post hoc pairwise comparisons were performed using estimated marginal means with Bonferroni correction.

Secreted factors (IL-6, E2 and P4) and cell proliferation (Population Doublings, PDs) are expressed as mean ± SD and were analysed using Linear Mixed-Effects Models (LMM) fitted on log(x+1)-transformed data, with cell preparation (MF-GCs vs. ENDO-GCs), FF condition (FF-MF vs. FF-ENDO) and treatment (DMSO vs. DIDS) as fixed effects, along with all two-way and three-way interactions. Individual biological replicates were included as a random intercept term to account for the paired DMSO/DIDS design. Fixed effects significance was assessed using Type III ANOVA with Satterthwaite’s approximation. Post hoc pairwise comparisons between DMSO and DIDS within each cell preparation × FF condition combination were performed using estimated marginal means with Bonferroni correction. Log-transformation was applied to meet model assumptions of normality and homoscedasticity. Percentage changes reported in the text are calculated from raw observed means for descriptive purposes only and are complementary to the *p*-values derived from log-transformed LMM. Model fit was evaluated by comparing the Akaike Information Criterion (AIC) between log-transformed and non-transformed models, with lower AIC values indicating better fit; the difference (ΔAIC) between the two models was used to quantify the improvement in fit. Normality of model residuals was assessed using the Shapiro–Wilk test. Where mild violations of normality were detected after log-transformation, LMMs were retained given their robustness to moderate departures from normality.

AE1 expression data, expressed as the ratio of MFI in DIDS-treated to DMSO-treated samples (MFI DIDS/MFI DMSO), were analysed using an LMM fitted on log-transformed ratio data, with cell preparation (MF-GCs vs. ENDO-GCs), FF condition (FF-MF vs. FF-ENDO), and their interaction as fixed effects, and individual sample identity as a random intercept term. Fixed effects significance was assessed using Type III ANOVA with Satterthwaite’s approximation. Post hoc pairwise comparisons between cell preparations and between treatment conditions were performed using estimated marginal means with Bonferroni correction. Additionally, one-sample *t*-tests on raw ratio values were applied to assess whether each group ratio differed significantly from 1 (no DIDS effect), with Bonferroni correction for multiple comparisons (*n* = 4 tests).

The DIDS-related effect on gene expression (*hSLC4A1-TM*, *hSLC4A1-N*, *hIL-6*, *CCND1*, *HSD3B1*, and *STAR*) was assessed by computing the ratio of values measured in DIDS-treated versus DMSO-treated samples (R_DIDS_/R_DMSO_). Ratios were analysed using LMM fitted on log-transformed data, with cell preparation (MF-GCs vs. ENDO-GCs) and FF condition (FF-MF vs. FF-ENDO) as fixed effects, along with their interaction, and individual sample identity as a random intercept term. Fixed effects significance was assessed using Type III ANOVA with Satterthwaite’s approximation. Post hoc pairwise comparisons between cell preparations and between FF conditions were performed using estimated marginal means (emmeans package) with Bonferroni correction. Additionally, one-sample *t*-tests on raw ratio values were applied to assess whether each group ratio differed significantly from 1 (no DIDS effect), with Bonferroni correction for multiple comparisons (*n* = 4 tests per gene).

For all analyses, statistical significance was set at *p* < 0.05.

## 3. Results

### 3.1. Patient Characterization

The 24 patients (*n*= 10 for the MF and *n* = 14 for the ENDO group, respectively) undergoing IVF procedures and enrolled in this study were separately considered and characterized.

There were no significant differences in age between the two groups, with a mean age of 31.4 ± 3.8 years in the MF group and 33.6 ± 3.4 years in the ENDO group (*p* = 0.156). No significant differences were observed in body mass index (BMI) (21.2 ± 2.5 vs. 21.4 ± 1.9 in the MF and ENDO groups, respectively) or in antral follicle count (AFC) (10.2 ± 3.6 vs. 9.6 ± 2.7) (Table 2).

The unequal group sizes reflect the natural distribution of diagnoses among eligible patients recruited during the study period and are not associated with any statistically significant differences in clinical or demographic parameters between groups, as reported in Table 2.

**Figure 1:**
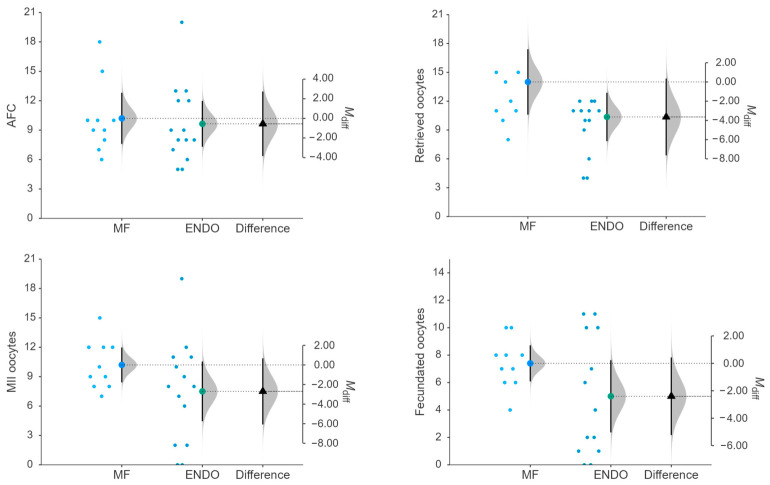
Gardner–Altman estimation plots comparing follicular and oocyte parameters (AFC, retrieved oocytes, MII oocytes, and fecundated oocytes) between MF and ENDO groups. (**Left** panels) of each graph show individual data points and group distributions (shaded areas); filled circles indicate group means; (**right** panels) show the mean difference (filled triangle) with 95% confidence interval. No significant differences were detected between groups (Student’s *t*-test). See also Table 2.

Figure 1 provides a more detailed visualization of the distribution of follicular and oocyte parameters in the two patient groups, compared with the summary values reported in Table 2.

Interestingly, no significant differences in fertilization rates were observed between the two groups (0.7 ± 0.2 vs. 0.7 ± 0.3 for the MF and ENDO groups, respectively), suggesting that controlled ovarian stimulation efficiently counteracted the potential negative impact of endometriosis on fertilization outcomes.

### 3.2. AE1 Expression

**Figure 2:**
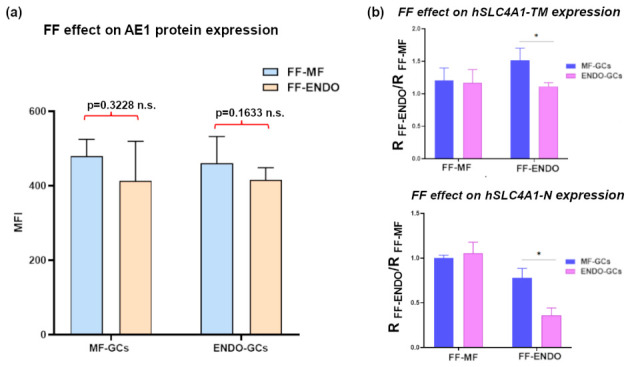
AE1 expression in MF-GCs and ENDO-GCs cultured in the presence of FF-MF or FF-ENDO. GCs were recovered and cultured as described in Section 2. Cells were then collected, washed in PBS, and divided into two aliquots. One aliquot (**a**) was fixed, permeabilized, and labeled with anti-AE1 antibody for flow cytometric analysis. Fluorescence intensity was recorded and expressed as mean fluorescence intensity (MFI) values ± SD from six independent experiments. Statistical significance was assessed using Student’s paired *t*-test (FF-MF vs. FF-ENDO within each cell preparation). n.s.: not significant. The second aliquot (**b**) was subjected to RNA extraction and quantitative real-time PCR analysis to evaluate *hSLC4A1-TM* and *hSLC4A1-N* gene expression levels. Data are expressed as mean ± SD from six independent experiments. * indicates *p* < 0.05.

Protein and gene expression of AE1 were evaluated in MF-GCs and ENDO-GCs cultured in FF-MF or FF-ENDO conditions (Figure 2). SLC4A1 expression was assessed using two independent primer sets targeting distinct regions of the same transcript. The *SLC4A1-N* primer pair amplifies a sequence within the N-terminal coding region [15,19], whereas the *SLC4A1-TM* primer pair amplifies a downstream sequence located within the transmembrane coding domain common to AE1 transcripts. The combined analysis of these two amplicons allowed confirmation of SLC4A1 transcript expression while assessing both the N-terminal and transmembrane coding regions. As shown in Figure 2, FF-ENDO treatment did not significantly affect AE1 protein expression (panel a), although a non-significant tendency toward reduction was observed, which should be interpreted with caution, while lower AE1 gene levels were detected in (panel b) compared with FF-MF conditions, suggesting that the endometriotic follicular microenvironment may influence AE1 expression in granulosa cells.

### 3.3. AE1 Localization

**Figure 3:**
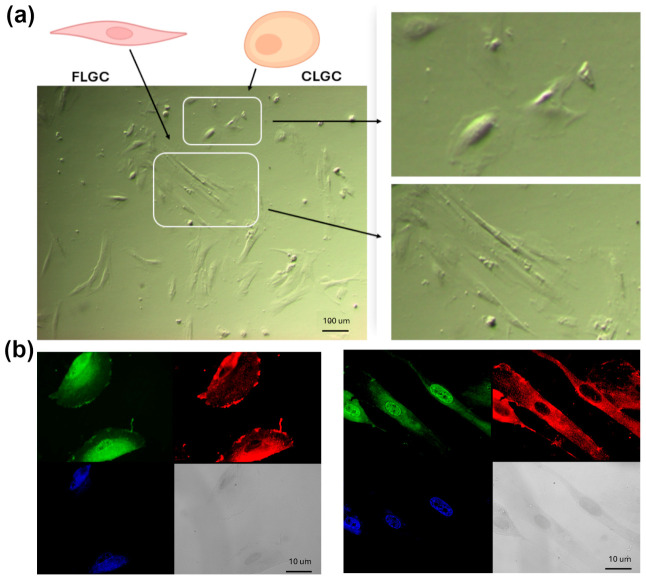
Identification and localization of the anion exchanger AE1 in CLGCs and FLGCs. (**a**) Contrast phase images of the GCs culture (on the left) before immunostaining with anti-AE1 antibodies. In the boxes on the right: fields showing a detail of the CLGC (top) and FLGC (bottom) subpopulations. (**b**) Fluorescent images of the CLGCs (on the left) and FLGCs (on the right) immunostained with anti-AE1 (green) or anti-FSHR (red) antibodies. Nuclei have been evidenced with Draq5 (blue). The fourth and last boxes at the right bottom show the light field. The figure is representative of 6 different experiments either in FF-MF or in ENDO-FF conditions.

MF-GCs and ENDO-GCs were separately treated with FF-MF or FF-ENDO for the ICC analysis on AE1 location (Figure 3). Results showed that there were interesting differences among morphological sub-populations (Figure 3a), especially between CLGCs and FLGCs, which showed distinct patterns of AE1 subcellular distribution, independently of the FF medium. In CLGCs, AE1 was uniformly distributed throughout the main cell body, particularly over and around the nucleus, which appeared fully covered, extending toward the cell periphery. In contrast, in FLGCs, AE1 was predominantly localized in the perinuclear region, gradually decreasing toward the cell extremities, where it maintained a punctate rather than diffuse pattern. In both cell types, FSHR expression was very similar, if not identical, and was not affected by treatment (Figure 3b).

### 3.4. AE1 Involvement in GCs Phenotype

**Figure 4:**
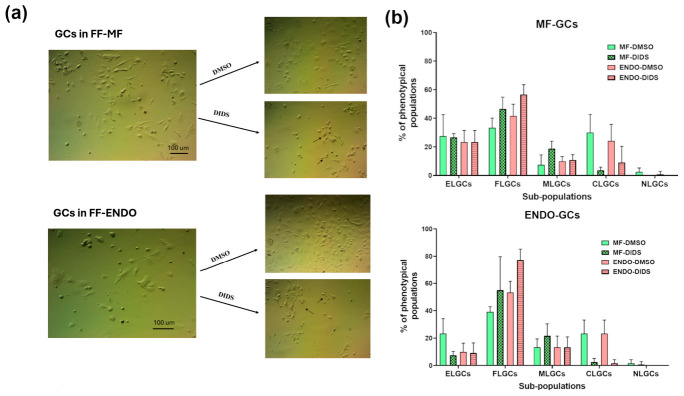
DIDS-related effect on GCs cultured in FF-MF and FF-ENDO. GCs from MF (MF-GCs) or ENDO (ENDO-GCs) patients were cultured in FF-MF or FF-ENDO in the presence of DIDS 100 µM or vehicle (DMSO) for 14 days. (**a**) Representative contrast-phase images of GC morphological subpopulations before (left) and after 14 days in the presence (black arrow DIDS) or absence (black arrow DMSO) of AE1 blocker. Scale bar = 10 μm. (**b**) Morphological assessment was performed by analyzing 10 fields per plate, as described in the Methods. A total of 500 cells per sample were evaluated and classified into ELGCs: epithelial-like granulosa cells; FLGCs: fibroblast-like granulosa cells; MLGCs: muscle-like granulosa cells; CLGCs: chondroblast-like granulosa cells; NLGCs: neuronal-like granulosa cells. For complete statistical analysis, see Figure 5.

**Figure 5:**
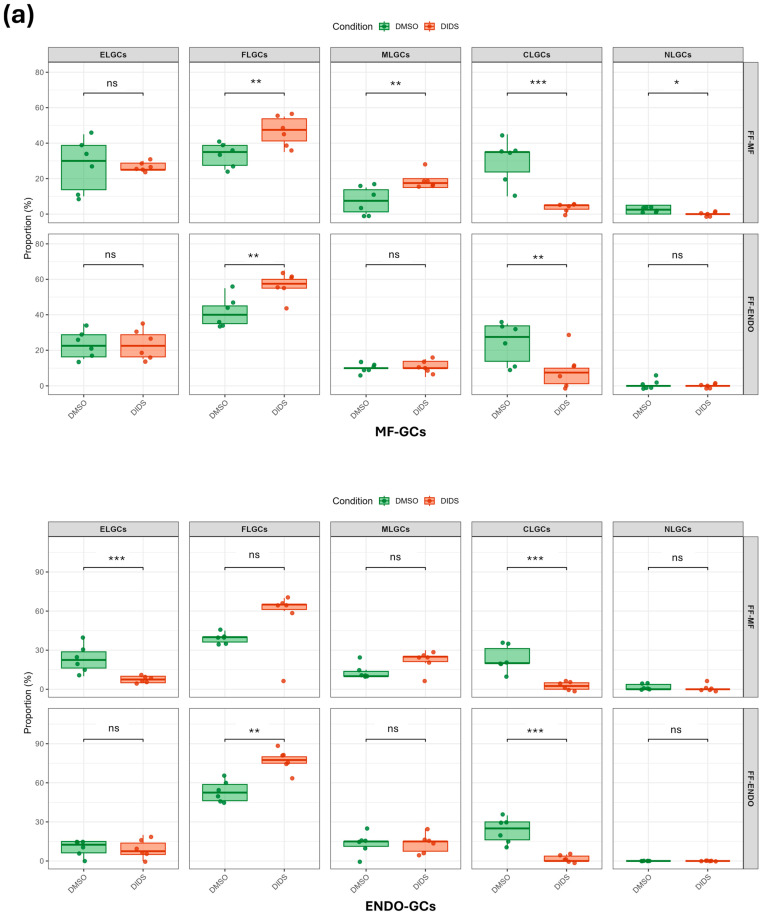

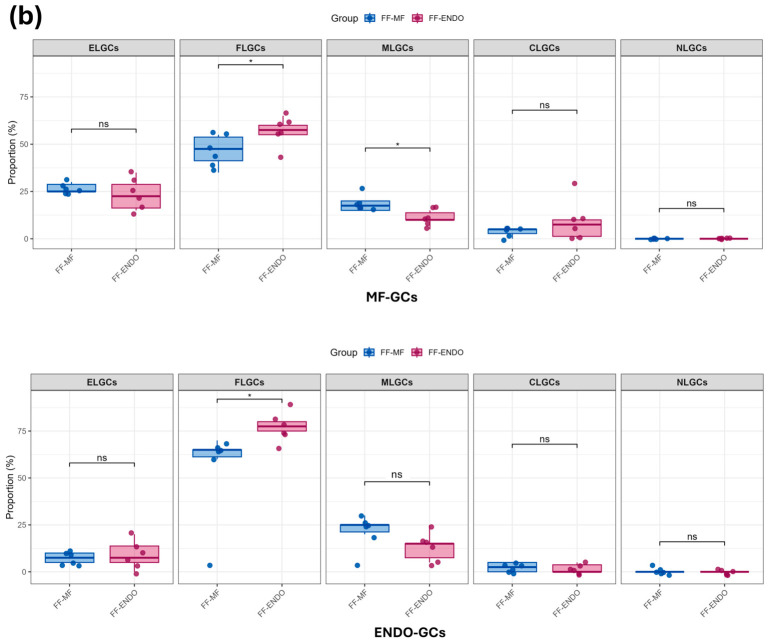
(**a**). Proportion of morphological subpopulations (ELGCs: epithelial-like; FLGCs: fibroblast-like; MLGCs: muscle-like; CLGCs: chondroblast-like; NLGCs: neuronal-like granulosa cells) in MF-GCs (**upper** panels) and ENDO-GCs (**lower** panels) cultured in FF-MF or FF-ENDO under DMSO or DIDS (100 μM) treatment. Brackets indicate paired comparisons between DMSO and DIDS within each FF condition (paired *t*-test, Bonferroni correction; * *p* < 0.05, ** *p* < 0.01, *** *p* < 0.001, ns: not significant). (**b**). Proportion of morphological subpopulations (ELGCs, FLGCs, MLGCs, CLGCs, NLGCs) in MF-GCs (**upper** panels) and ENDO-GCs (**lower** panels) under DIDS (100 μM) treatment: comparison between FF-MF and FF-ENDO conditions. Brackets indicate pairwise post hoc comparisons based on model-estimated marginal means (Bonferroni correction; * *p* < 0.05, ns: not significant).

To assess the involvement of AE1 in GCs phenotype, MF-GCs and ENDO-GCs were cultured in the presence or absence of 4,4′-Diisothiocyanatostilbene-2,2′-disulfonic acid (DIDS: SIGMA-Aldrich, Saint Louis, MO 63103, USA), a well-established pharmacological inhibitor of anion exchangers, particularly AE1 [16,20]. DIDS binds covalently to lysine residues located within the membrane domain of AE1, thereby blocking chloride/bicarbonate exchange activity and altering intracellular pH regulation and ion transport mechanisms [21,22]. Based on previous studies investigating AE1-associated anion exchange activity in reproductive and other cellular models, DIDS was used at a concentration of 100 μM [20,23,24]. The experimental plan, together with the DIDS-related effects on GCs, is reported in Figure 4. Figure 5 provides a more detailed visualization of the distribution of GCs subpopulations under the different conditions.

GCs were classified into distinct morphological categories adopted exclusively as descriptive morphological patterns to facilitate the evaluation of culture heterogeneity and treatment-induced morphological changes and growth characteristics [4]. Fibroblast-like GCs (FLGCs) displayed an elongated, spindle-shaped morphology with tapered ends and long cytoplasmic extensions, frequently establishing contact with neighboring cells. Epithelial-like GCs (ELGCs) exhibited a typical cobblestone appearance, growing as compact colonies of polygonal cells with close cell-to-cell apposition [25,26]. Chondroblast-like GCs (CLGCs) appeared as rounded or disc-shaped cells surrounded by a homogeneous pericellular matrix-like area. Muscle-like GCs (MLGCs) showed an elongated spindle-shaped morphology with a broader central region and tapered extremities. Neuronal-like GCs (NLGCs) were characterized by elongated cell bodies with thin, extended cytoplasmic processes. These categories were adopted exclusively as descriptive morphological patterns to facilitate the evaluation of culture heterogeneity and treatment-induced morphological changes.

Figure 5 provides a more detailed visualization of the distribution of GC subpopulations under the different conditions.

#### 3.4.1. Effect of DIDS Treatment on GCs Morphological Sub-Populations

DIDS treatment significantly altered the proportion of morphologically distinct GC subpopulations in both MF-GCs and ENDO-GCs, although with partially distinct patterns between the two preparations (Figure 5a).

FLGCs increased significantly under DIDS in both preparations, with a significant main effect of both treatment (F = 12.63, *p* = 0.002) and FF condition (F = 10.61, *p* = 0.004) in ENDO-GCs: in MF-GCs, this was observed under both FF-MF (*p* = 0.0044) and FF-ENDO (*p* = 0.0019) conditions, while in ENDO-GCs, the increase reached significance under FF-ENDO (+24.0%, *p* = 0.009), with a comparable but non-significant trend under FF-MF (*p* = 0.064), likely due to the influence of an outlier reducing test power. Under DIDS, FF-ENDO showed significantly more FLGCs than FF-MF in ENDO-GCs (*p* = 0.013), a difference already presented as a trend under DMSO (*p* = 0.094).

CLGCs showed the most consistent response across both preparations, with a significant reduction following DIDS treatment. In MF-GCs, this was significant under both FF-MF (*p* < 0.0001) and FF-ENDO (*p* = 0.004) conditions. Similarly, in ENDO-GCs, CLGCs were significantly reduced under both FF-MF (−20.8%, *p* = 0.0001) and FF-ENDO (−21.7%, *p* < 0.0001), with a strong main effect of treatment (F = 61.83, *p* < 0.0001) and no significant FF condition effect or interaction, indicating a uniform response across both experimental groups.

MLGCs responded differently between preparations: in MF-GCs, a significant reduction was observed under FF-MF (*p* = 0.0012), with a significant treatment × FF condition interaction (*p* = 0.017) indicating a group-specific response, while in ENDO-GCs MLGCs were not significantly affected by DIDS under either condition (FF-MF *p* = 0.080, FF-ENDO *p* = 1.000).

ELGCs showed a preparation-specific response: in MF-GCs, they remained unaffected under both FF conditions (*p* = 0.863), whereas in ENDO-GCs, DIDS induced a significant reduction exclusively under FF-MF (−15.8%, *p* = 0.0007), with FF-ENDO remaining unaffected (*p* = 0.825), resulting in a significant treatment × FF condition interaction (F = 8.21, *p* = 0.012).

NLGCs were significantly reduced in MF-GCs under FF-MF (*p* = 0.023), but were unaffected in ENDO-GCs under either condition (FF-MF *p* = 0.341, FF-ENDO *p* = 1.000).

When comparing FF-MF and FF-ENDO under DIDS treatment (Figure 5b), FF-ENDO showed a significantly higher proportion of FLGCs and lower proportion of MLGCs than FF-MF in MF-GCs (*p* = 0.024 and *p* = 0.013, respectively), while in ENDO-GCs FF-ENDO showed significantly more FLGCs than FF-MF (*p* = 0.013).

#### 3.4.2. Effect of DIDS Treatment on AE1 Expression

**Figure 6:**
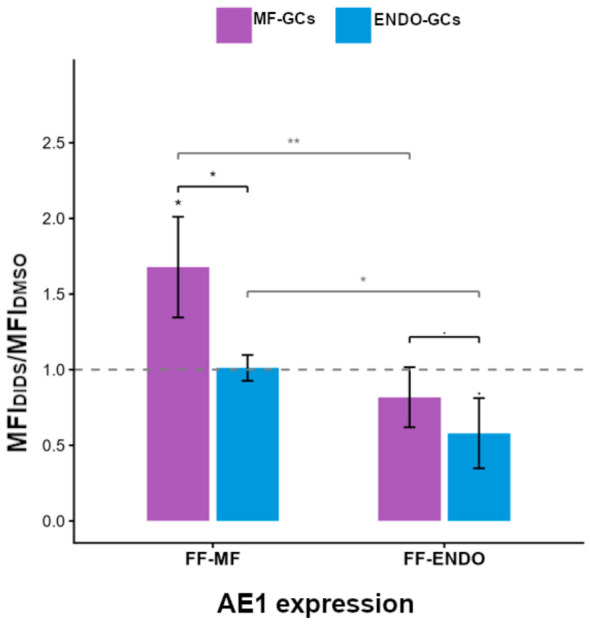
DIDS (100 μM)-related effect on AE1 protein expression. Fluorescence is expressed as the MFI of the DIDS-treated sample/MFI of the DMSO control. Bar graphs show mean ± SD of the AE1 expression ratio (MFI DIDS/MFI DMSO) for MF-GCs (purple) and ENDO-GCs (blue) under FF-MF and FF-ENDO experimental conditions (*n* = 5 per group). The dashed line indicates a ratio of 1 (no DIDS effect). Statistical analysis: LMM on log-transformed ratio data. Symbols above individual bars indicate significant deviation from ratio = 1 (one-sample *t*-test, Bonferroni-corrected). Black brackets: significant differences between MF-GCs and ENDO-GCs within each condition; grey brackets: significant differences between FF-MF and FF-ENDO within each cell line (post hoc comparisons on model-estimated means, Bonferroni-corrected). Significance levels: •, *p* < 0.10; *, *p* <0.05; **, *p* < 0.01.

The DIDS-related effect on AE1 expression was assessed by computing the ratio of MFI values measured in DIDS-treated versus DMSO-treated samples (MFI DIDS/MFI DMSO) for each cell preparation (MF-GCs and ENDO-GCs) under FF-MF and FF-ENDO conditions (Figure 6)**.** A ratio > 1 indicates DIDS-induced upregulation, whereas a ratio < 1 indicates downregulation relative to vehicle control.

LMM analysis revealed the significant main effects of both cell preparations (F_1,8_ = 9.15, *p* = 0.016) and Treatment 1 (F_1,8_ = 21.87, *p* = 0.002), with no significant interaction (*p* = 0.827), indicating that the effect of DIDS on AE1 expression differed between cell preparations and between FF conditions, but in a consistent, additive manner.

Under FF-MF conditions, DIDS significantly increased AE1 expression in MF-GCs (ratio = 1.68 ± 0.33, *p* = 0.042 vs. ratio of 1), while no significant effect was observed in ENDO-GCs (ratio = 1.01 ± 0.09, *p* = 1.000). Under FF-ENDO conditions, both cell preparations showed ratios below 1, suggesting a trend toward DIDS-induced downregulation of AE1, which, however, did not reach statistical significance in either ENDO-GCs (ratio = 0.58 ± 0.23, *p* = 0.062) or MF-GCs (ratio = 0.82 ± 0.20, *p* = 0.440). Post hoc comparisons confirmed that AE1 ratios were significantly higher under FF-MF than FF-ENDO conditions in both MF-GCs (*p* = 0.0085) and ENDO-GCs (*p* = 0.014), suggesting a context-dependent modulation of AE1 by DIDS.

#### 3.4.3. DIDS Effect on Interleukin Secretion, Hormonal Production and Cell Proliferation in GCs

**Figure 7:**
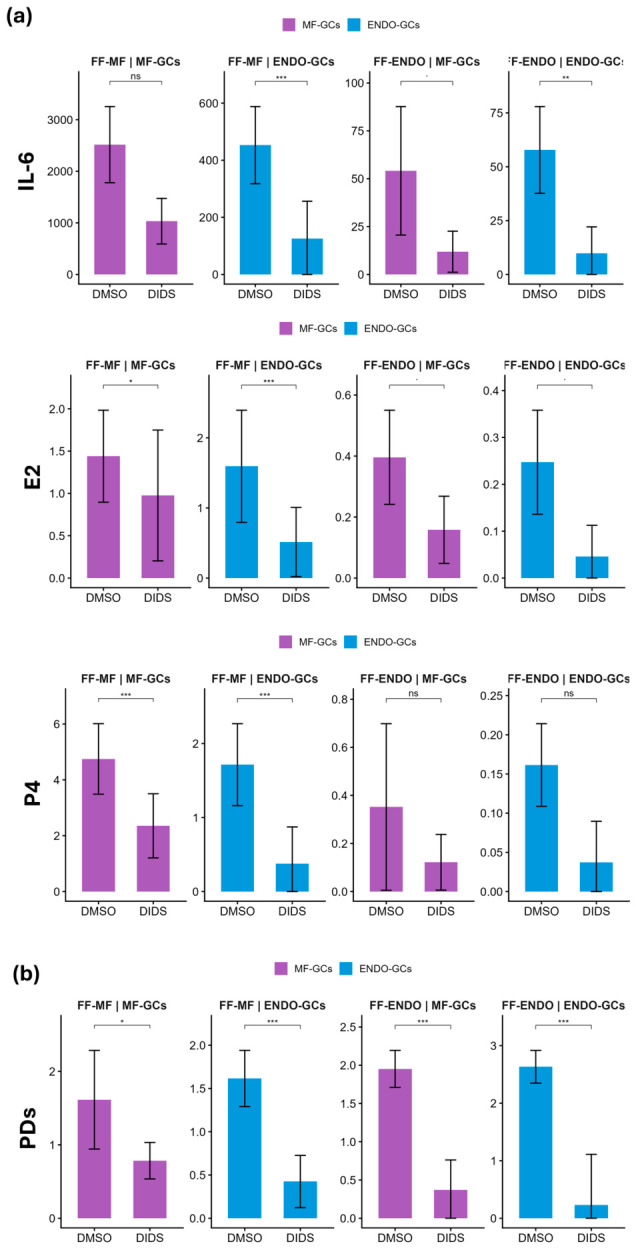
Evaluation of DIDS (100 μM) effects on (**a**) IL-6, E2 and P4 secretion and (**b**) cell proliferation (PDs) in MF-GCs (purple) and ENDO-GCs (blue) treated with DMSO or DIDS under FF-MF and FF-ENDO conditions. Data are mean ± SD. Statistical analysis: LMM on log-transformed data, Bonferroni correction. ns: *p* ≥ 0.10; ‘•’: *p* < 0.10; *: *p* < 0.05; **: *p* < 0.01; ***: *p* < 0.001. For model diagnostics, see the Results Section 3.4.3.

In addition to its effects on ion transport-related gene expression, DIDS also affected general physiological functions in GCs (Figure 7). As previously reported, FF strongly modulates GCs function: FF-ENDO negatively affects IL-6, E2, and P4 expression at both gene and protein levels, while increasing the expression of *CCND1*, a gene involved in cell proliferation [5].

In MF-GCs incubated with FF-MF (Figure 7a), DIDS treatment significantly reduced E2 (estimate = 0.241, *p* = 0.032) and P4 (estimate = 0.591, *p* < 0.001) secretion, with mean decreases of 46% and 48%, respectively (calculated from raw observed means), while IL-6 reduction (58%) did not reach statistical significance (*p* = 0.300). These inhibitory effects were further pronounced when MF-GCs were exposed to FF-ENDO in the presence of DIDS, although none of the reductions reached statistical significance under these conditions (IL-6: *p* = 0.065; E2: *p* = 0.090; P4: *p* = 0.236), and, therefore, no conclusion can be drawn regarding the effect of FF-ENDO on DIDS-mediated inhibition in MF-GCs.

In ENDO-GCs, the already limited IL-6 expression observed under FF-MF control conditions (DMSO) was further significantly reduced by DIDS (74%; raw means; estimate = 3.025, *p* < 0.001), as were E2 (69%; raw means; estimate = 0.542, *p* < 0.001) and P4 (78%; raw means; estimate = 0.712, *p* < 0.001). An additional reduction was observed when ENDO-GCs were simultaneously exposed to FF-ENDO and DIDS, with IL-6 significantly reduced by 78% (raw means; estimate = 2.619, *p* = 0.002), while E2 and P4 did not reach statistical significance under FF-ENDO conditions (E2: *p* = 0.062; P4: *p* = 0.336). Cell proliferation was broadly and consistently impaired by DIDS across all experimental groups. In MF-GCs, cell replication decreased by more than 50% under FF-MF conditions (estimate = 0.367, *p* = 0.023) and was even more strongly reduced under FF-ENDO (estimate = 0.803, *p* < 0.001). ENDO-GCs, which normally exhibit high proliferative activity under FF-ENDO compared with FF-MF [4,5], showed significant reductions in PDs under both FF-MF (estimate = 0.620, *p* < 0.001) and FF-ENDO (estimate = 0.986, *p* < 0.001) conditions, becoming almost completely unable to proliferate when simultaneously exposed to FF-ENDO and DIDS. Model diagnostics confirmed the adequacy of log-transformed LMMs for all outcomes. The Shapiro–Wilk test on model residuals showed mild violations of normality for P4 (W = 0.946, *p* = 0.027) and PDs (W = 0.947, *p* = 0.034), and a borderline result for IL-6 (W = 0.959, *p* = 0.091); LMMs were retained given their robustness to moderate departures from normality and the substantially better fit of log-transformed models over untransformed ones (ΔAIC: 92.65, 66.2, and 497.42 for P4, PDs, and IL-6, respectively).

#### 3.4.4. DIDS Effect on GCs Gene Expression Under Different Conditions

**Figure 8:**
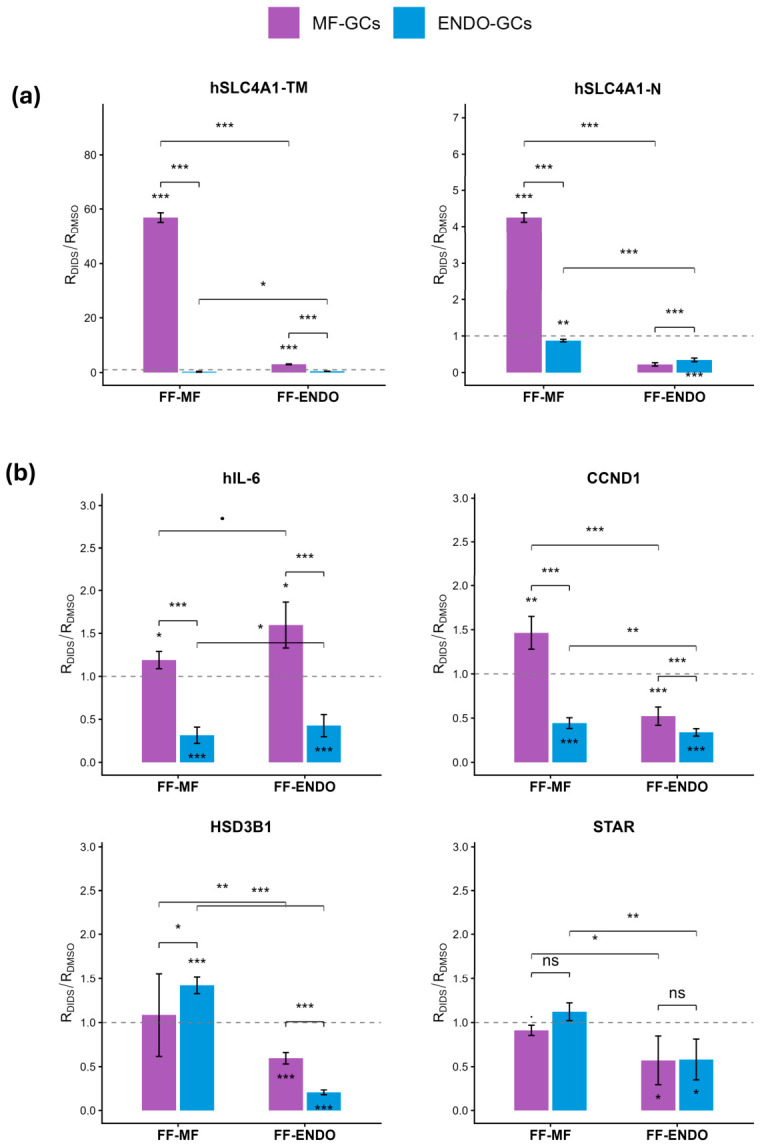
Modulation of (**a**) *hSLC4A1-TM* and *hSLC4A1-N* and (**b**) hIL-6, CCND1, HSD3B1 and STAR gene expression in MF-GCs (violet) and ENDO-GCs (blue) cultured with FF-MF or FF-ENDO in the presence of DIDS (100 μM) or DMSO. Data are expressed as DIDS/DMSO expression ratios (mean ± SD, *n* = 6 independent experiments per group). The dashed horizontal line indicates a ratio of 1 (no DIDS effect). Statistical analysis: LMM on log-transformed data, Bonferroni correction. Asterisks above/below bars: one-sample *t*-test vs. ratio = 1. Brackets: post hoc comparisons between groups. ns: *p* ≥ 0.10; •: *p* < 0.10; *: *p* < 0.05; **: *p* < 0.01; ***: *p* < 0.001.

To better investigate the potential effects of DIDS on GC gene expression (Figure 8), quantitative real-time PCR was performed on GCs cultured in FF-MF or FF-ENDO in the presence of DIDS (100 μM) or vehicle (DMSO). Gene expression levels of AE1 (*hSLC4A1-TM* and *hSLC4A1-N*) were evaluated (Figure 8a), together with IL-6 (*hIL6*), cyclin D1 (*CCND1*), hydroxy-Δ5-steroid dehydrogenase, 3β- and steroid Δ-isomerase 1 (*HSD3B1*), and steroidogenic acute regulatory protein (*STAR*) (Figure 8b).

The DIDS-related effect on gene expression was assessed by computing the ratio of values measured in DIDS-treated versus DMSO-treated samples (R_DIDS_/R_DMSO_) for each cell line (MF-GCs and ENDO-GCs) under FF-MF and FF-ENDO conditions. A ratio > 1 indicates DIDS-induced upregulation, whereas a ratio < 1 indicates downregulation relative to vehicle control. Statistical analysis was performed using an LMM fitted on log-transformed ratio data, with cell preparation and FF condition as fixed effects, their interaction, and individual sample identity as a random intercept term (see Section 2.11). One-sample *t*-tests against ratio = 1 were applied with Bonferroni correction (*n* = 4 tests per gene).

In MF-GCs cultured with FF-MF, DIDS markedly increased the expression of both *hSLC4A1-TM* (ratio = 56.83, *p* < 0.001) and *hSLC4A1-N* (ratio = 4.25, *p* < 0.001), with *hSLC4A1-TM-* showing the strongest induction, likely reflecting a compensatory response to DIDS-mediated -chemical blockade. A significant increase in *hIL-6* (ratio = 1.19, *p* = 0.023) and a modest upregulation of *CCND1* (ratio = 1.47, *p* = 0.007) were also observed, while *HSD3B1* remained substantially unchanged (*p* = ns) and *STAR* showed only a trend toward decrease (*p* = 0.053).

Conversely, when MF-GCs were cultured in FF-ENDO, DIDS was associated with a generalized reduction in gene expression. In particular, *hSLC4A1-N, CCND1, HSD3B1*, and *STAR* expression levels were significantly decreased (all *p* ≤ 0.050), whereas *hSLC4A1-TM* remained upregulated (ratio = 3.00, *p* < 0.001) and *hIL-6* further increased (ratio = 1.60, *p* = 0.011).

ENDO-GCs showed a consistent inhibitory response to DIDS under both conditions. Under FF-MF, most genes were significantly downregulated or showed a decreasing trend, with the most pronounced effects on *hSLC4A1-TM* (ratio = 0.27, *p* < 0.001) and *hIL-6* (ratio = 0.31, *p* < 0.001); *hSLC4A1-N* was also significantly reduced (ratio = 0.87, *p* = 0.001). Notably, HSD3B1 showed a significant upregulation under FF-MF conditions (ratio = 1.42, *p* < 0.001), suggesting a possible compensatory steroidogenic response in ENDO-GCs when AE1 is inhibited in a non-endometriotic microenvironment. Under FF-ENDO, DIDS caused a marked downregulation of all analyzed genes, including *hSLC4A1-TM* (ratio = 0.44, *p* < 0.001), *hSLC4A1-N* (ratio = 0.34, *p* < 0.001), *hIL-6* (ratio = 0.43, *p* < 0.001), *CCND1* (ratio = 0.44, *p* < 0.001), *HSD3B1* (ratio = 0.20, *p* < 0.001), and *STAR* (ratio = 0.58, *p* = 0.027).

LMM confirmed significant CellLine × Treatment interactions for *hSLC4A1-TM hSLC4A1-N*, *CCND1*, and *HSD3B1* (all *p* < 0.001), reflecting the differential response of the two cell lines to DIDS depending on the follicular microenvironment. For *hIL-6* and *STAR*, no significant interaction was detected, indicating a more uniform pattern across conditions. It should be noted that these changes were assessed exclusively at the transcript level; protein-level validation of CCND1, STAR, and HSD3B1 was not performed and is required before functional conclusions can be drawn.

These findings are consistent with the involvement of DIDS-sensitive anion exchange activity, with AE1 representing the most characterized target of DIDS [20,21] although the contribution of other DIDS-sensitive transporters cannot be excluded. Notably, *hSLC4A1-TM* and *hSLC4A1-N* were the only gene transcripts markedly upregulated. Notably, *hSLC4A1-TM* and *hSLC4A1-N* were the only gene transcripts markedly upregulated following DIDS exposure, particularly in MF-GCs cultured with FF-MF, likely reflecting a compensatory cellular response aimed at counteracting the chemical blockade of AE1 activity. In contrast, the concomitant reduction in genes involved in proliferation, inflammation, and steroidogenesis, reflecting the corresponding decrease in the expression of IL-6, E2 and P4 and the cellular replication (PDs), indicates that AE1 inhibition exerts a profound negative impact on granulosa cell homeostasis. This effect was especially evident in FF-ENDO conditions and in ENDO-GCs, where the endometriotic follicular microenvironment appears to exacerbate the cellular stress associated with AE1 functional impairment, ultimately leading to a generalized suppression of transcriptional activity. However, under FF-MF conditions, ENDO-GCs showed a significant upregulation of HSD3B1 (ratio = 1.42, *p* < 0.001), suggesting that a partial compensatory steroidogenic response may be retained when the endometriotic microenvironment is absent.

## 4. Discussion

For the first time, we provide preliminary evidence for the expression of AE1 in human GCs and suggest its potential role in the regulation of GC physiology. Our findings show that DIDS-sensitive anion exchange activity is associated with multiple cellular processes, including cell proliferation, morphology, inflammatory response, and steroidogenic activity, as assessed by morphological criteria, influencing both protein production and gene transcription. In particular, modulation of AE1 activity by DIDS markedly altered the expression of transport-related genes (*hSLC4A1-TM* and *hSLC4A1-N*), proliferation-(PDs and *CCND1*), inflammation-associated markers (IL-6), as well as key steroidogenic regulators (E2 and P4 with *HSD3B*1 and *STAR* transcripts). Moreover, we demonstrated that the follicular microenvironment strongly influences AE1-dependent responses, with FF-ENDO conditions exacerbating the detrimental effects of AE1 inhibition, especially in ENDO-GCs. Notably, our preliminary results suggest that AE1 activity may contribute to granulosa cell homeostasis, since both MF-GCs and ENDO-GCs attempted to counteract DIDS-mediated inhibition by increasing AE1 expression, particularly under FF-MF conditions. Interestingly, ENDO-GCs also showed a significant upregulation of HSD3B1 under FF-MF conditions, further supporting the existence of residual compensatory capacity in these cells when not exposed to the endometriotic follicular microenvironment. This compensatory response was evident both at the protein and transcript levels and was especially pronounced in MF-GCs. Interestingly, among the analyzed genes, *hSLC4A1-TM* was by far the most strongly upregulated transcript following DIDS exposure, further supporting the hypothesis that GCs actively respond to AE1 functional blockade by enhancing AE1 synthesis in an attempt to restore exchanger activity. In contrast, under FF-ENDO conditions, neither MF-GCs nor ENDO-GCs were able to mount an effective compensatory response against DIDS inhibition, resulting in a generalized suppression of gene expression and cellular activity.

The anion exchanger 1 (AE1), is a multifunctional transmembrane glycoprotein belonging to the solute carrier 4 (SLC4) family of bicarbonate transporters [16,21]. AE1 is primarily expressed in erythrocytes and renal α-intercalated cells, where it mediates the electroneutral exchange of chloride (Cl^−^) and bicarbonate (HCO_3_^−^), thereby contributing to intracellular pH regulation, CO_2_ transport, osmotic balance, and cellular redox homeostasis [15]. Beyond its transporter activity, AE1 acts as a structural scaffold protein interacting with ankyrin, spectrin, and glycolytic enzymes, thus linking membrane stability to cellular metabolism and oxidative stress responses [27]. Alterations in AE1 phosphorylation state and membrane organization have been associated with oxidative damage, inflammation, and impaired antioxidant defenses [28,29,30].

DIDS inhibits AE1 through a two-step mechanism. First, DIDS rapidly binds AE1 through a reversible electrostatic interaction. Subsequently, it forms a slower irreversible covalent bond with a lysine residue of AE1, identified in human AE1 as Lys539 (K539), located near the extracellular region of transmembrane domain 5 [21,22].

Interestingly, increasing evidence suggests that oxidative stress is a central feature of endometriosis [31,32] and contributes to both systemic and follicular dysfunction [33]. In women with endometriosis, elevated levels of reactive oxygen species (ROS), lipid peroxidation products, and oxidative DNA damage have been detected in serum, follicular fluid [32,34,35], and ovarian tissue, correlating with impaired oocyte quality and infertility. In particular, oxidative imbalance within the follicular microenvironment may alter granulosa cell physiology and disrupt the biochemical composition of follicular fluid, ultimately compromising folliculogenesis and embryo competence [34,36].

Our previous studies demonstrated that AE1/Band 3 is highly sensitive to the oxidative milieu associated with endometriosis, showing increased tyrosine phosphorylation, glutathione depletion, and enhanced susceptibility to oxidative injury in erythrocytes from affected women [29,31]. In light of the present findings, it is conceivable that a similar condition may also occur in granulosa cells. Indeed, chronic exposure of ENDO-GCs to oxidative and inflammatory stress could continuously affect AE1 structure and function, ultimately reducing the cellular capacity to adequately regulate or replenish the exchanger. This hypothesis may explain why ENDO-GCs exhibited a markedly weaker compensatory response to DIDS-mediated AE1 blockade compared with MF-GCs, despite the evident requirement for AE1 activity in maintaining cellular homeostasis.

Moreover, the FF-ENDO microenvironment itself, known to be enriched in ROS, lipid peroxidation products, inflammatory cytokines, and oxidatively modified molecules [34], may further compromise AE1 functionality and membrane stability. Under these conditions, DIDS-induced AE1 blockade may therefore act on a transporter already functionally impaired or oxidatively damaged, exacerbating cellular stress and limiting the ability of GCs to activate compensatory transcriptional programs. The reduced *hSLC4A1-TM* and *hSLC4A1-N* transcription observed in FF-ENDO conditions may consequently derive from an impairment of redox-sensitive signaling pathways and transcriptional machinery, mitochondrial dysfunction, or from a generalized metabolic shutdown induced by persistent oxidative stress and intracellular acid–base imbalance.

The observed reduction in estradiol (E2) secretion following DIDS treatment deserves particular attention. E2 biosynthesis in granulosa cells depends on aromatase (CYP19A1), whose activity is closely linked to mitochondrial function and intracellular pH homeostasis [35,36]. Since DIDS-sensitive anion exchange contributes to the regulation of intracellular acid-base balance, its inhibition may alter the ionic environment required for optimal mitochondrial function [15,37]. As a speculative but biologically plausible consequence, impaired mitochondrial activity could in turn reduce cholesterol transport to the inner mitochondrial membrane, a rate-limiting step in steroidogenesis mediated by STAR [38], thereby limiting substrate availability for estrogen biosynthesis [39,40]. Although these mechanisms were not directly investigated in the present study, they represent biologically plausible hypotheses that could account for the reduction in E2 secretion observed following DIDS treatment, and warrant direct experimental investigation. Likewise, the increased HSD3B1 expression detected in ENDO-GCs cultured under FF-MF conditions may reflect a compensatory transcriptional response, although confirmation at the protein and functional levels will be required.

More generally, inhibition of DIDS-sensitive anion exchange activity may influence granulosa cell physiology through alterations in intracellular pH homeostasis. Because pH regulation is closely linked to mitochondrial metabolism, steroidogenesis, vesicular trafficking, and intracellular signaling [37,39,40,41], disruption of these processes could contribute to the changes observed in inflammatory signaling, steroid hormone secretion, and cell morphology. However, since intracellular pH, ion transport, mitochondrial function, and oxidative stress were not directly assessed, these mechanistic interpretations should be regarded as hypotheses requiring further experimental validation. In particular, future studies combining direct measurements of anion exchange activity with genetic approaches will be necessary to define the specific contribution of AE1 relative to other DIDS-sensitive transporters.

It should also be emphasized that the present study investigated two independent experimental variables: the follicular fluid microenvironment (FF-MF versus FF-ENDO) and the granulosa cell background (MF-GCs versus ENDO-GCs). Their individual and interactive effects were evaluated using linear mixed-effects models. Nevertheless, disentangling the relative contribution of the follicular microenvironment from intrinsic cellular alterations remains challenging in this experimental model and warrants further investigation.

Accordingly, the present study does not resolve the specific mechanistic pathway linking AE1 to the observed cellular responses, and further studies will be required to clarify its contribution relative to other DIDS-sensitive transporters and to define the underlying mechanisms.

## 5. Conclusions

The present exploratory in vitro pilot study provides preliminary evidence that DIDS-sensitive anion exchange activity contributes to granulosa cell homeostasis and may participate in the altered follicular physiology associated with endometriosis. The study was designed to identify biological mechanisms shared across the endometriotic follicular environment while minimizing the contribution of individual confounding factors. Because AE1 expression was demonstrated in human granulosa cells at both the transcript and protein levels, AE1 represents a biologically plausible candidate underlying these observations. However, the present findings should be regarded as hypothesis-generating and require confirmation in larger, independent cohorts.

The main limitations of this study include its in vitro design, the use of a pharmacologically non-selective inhibitor, and the absence of direct functional measurements of anion exchange activity and intracellular pH. Therefore, the specific contribution of AE1 to the observed cellular responses cannot be conclusively established. Future studies employing complementary genetic, biochemical, and functional approaches will be required to define the role of AE1 and other DIDS-sensitive transporters in granulosa cell physiology. In particular, further investigation of the relationship between anion exchange activity, intracellular pH regulation, mitochondrial metabolism, steroidogenic signaling, and oxidative stress within the endometriotic follicular microenvironment will be essential to elucidate the underlying mechanisms. Protein-level validation of individual transcriptomic targets such as CCND1, STAR, and HSD3B1 was not performed and represents an additional limitation of the present study.

Overall, this exploratory study provides a basis for future investigations combining genetic, biochemical, and functional approaches to clarify the contribution of AE1 and DIDS-sensitive anion exchange activity to granulosa cell physiology and the pathophysiology of endometriosis.

## Figures and Tables

**Table 1:** Sequence of oligonucleotides.

Gene	Oligonucleotide (5’–3’)	
	Forward	Reverse
*hIL6*	ACTCACCTCTTCAGAACGAATTG	CCATCTTTGGAAGGTTCAGGTTG
*HSD3B1*	GTCTTCGGTGTCACTCACAGAG	CTGGTGTAGATGAAGACTGGCAC
*hSLC4A1-N*	TGACATCACAGATGCATTCAG	ATCTGGTTCCGGGTCTTTTC
*hSLC4A1-TM*	CTGCTTGTGGTCGGCTTCTCAG	ACCAGCAGGATGAGCCAGAAGC
*STAR*	TCTCTACAGTGACCAGGAGC	GAACACCTTGCCCACATCTG
*CCND1*	TCTACACCGACACTCCATCCG	TCTGGCATTTTGGAGAGGAAGTG
*ACTB*	CCAAGGCCAACCGCGAGAAGAT	AGGGTACATGGTGGTGCCGCCA

**Table 2:** Patients’ parameters. Total FSH dose (expressed in international units, IU) administered during COS; Age and Body Mass Index (BMI), were considered for the evaluation of ovarian sensitivity, together with the COS outcome in follicle number (antral follicle count, AFC), total retrieved oocytes, MII oocytes (Metaphase II oocytes, as inseminated oocytes) and the number of fecundated oocytes. The fertilization rate was evaluated as the number of fecundated oocytes divided by the number of mature oocytes (MII). Additional clinical characteristics of patients are presented in Table S1.

	MF *n* = 10	ENDO *n* = 14	*p * ^†^
Total FSH (IU)	2135.0 ± 732.7	2617.1 ± 829.5	0.155
Age (years)	31.4 ± 3.8	33.6 ± 3.4	0.156
BMI (kg/m^2^)	21.2 ± 2.5	21.4 ± 1.9	0.864
AFC	10.2 ± 3.6	9.6 ± 4.1	0.733
Retrieved oocytes	14.0 ± 4.8	10.4 ± 4.4	0.065
MII oocytes	10.2 ± 2.5	7.5 ± 5.3	0.148
Fecundated oocytes	7.4 ± 1.8	5.0 ± 4.4	0.119
Fertilization rate	0.7 ± 0.2	0.7 ± 0.3	0.441

^†^ Student’s *t* test for unpaired data.

## Data Availability

The original contributions presented in this study are included in the article/Supplementary Materials. Further inquiries can be directed to the corresponding author.

## Supplementary Materials

The following supporting information can be downloaded at https://www.mdpi.com/article/10.3390/biom16071004/s1. Table S1: Additional clinical characteristics of patients.

## Abbreviations

The following abbreviations are used in this manuscript:

GCs	Granulosa Cells
AE1	Anion Exchanger 1
FF	Follicular Fluid
FSH	Follicle stimulating hormone
E2	Estradiol
LH	Luteinizing Hormone
P4	Progesterone
MF	Male Factor
ENDO	Endometriosis
ECM	Extracellular Matrix
FF-CM	Follicular Fluid added Culture Medium
FF-MF	Culture Medium with follicular fluid from MF patients
AMH	Anti-Müllerian Hormone
DIDS	4,4′-diisothiocyanatostilbene-2,2′-disulfonic acid
FF-ENDO	Culture Medium with follicular fluid from ENDO patients
CCM	Conditioned Culture Medium
