# Supplementary material for: Expression of Anion Exchanger 1 (AE1) and Its Potential Involvement in Human Granulosa Cell Physiology: An In Vitro Pilot Study
Source: Biomolecules. 2026 Jul 9;16(7):1004. doi: 10.3390/biom16071004 (PMC13407237; doi:10.3390/biom16071004)
Supplement: Supplementary file 1 [file biomolecules-16-01004-s001.zip › biomolecules-4370572-supplementary.pdf]

Table S1. Additional clinical characteristics of patients. ENDO patients showed significantly lower follicle number and FOI, consistent with reduced ovarian reserve. Serum hormonal profiles differed markedly between groups, with ENDO patients showing significantly higher FSH and E2 levels, lower LH, and comparable P4. Strikingly, serum IL-6 levels were markedly elevated in ENDO patients compared to MF controls ($p < 0.001$), confirming the pro-inflammatory systemic environment associated with endometriosis. FORT did not differ significantly between groups.

**Parameter**	**MF**<br>*n*=10	**ENDO**<br>n=14	***p*†**
FORT	1.7±1.3	1.5 ± 0.5	0.535
FOI	1.7 ± 0.4	1.3 ± 0.4	**0.045**
Follicles (n)	10.2 ± 3.6	6.4 ± 1.6	**0.002**
FSH (IU/L)	9.6± 3.2	28.3 ± 16.9	**0.003**
LH (IU/L)	6.4 ± 3.3	3.9 ± 1.3	**0.036**
E2 (µmol/L)	1.6 ± 0.6	4.1± 2.0	**0.002**
P4 (µmol/L)	56.8 ± 17.5	54.7 ± 19.8	0.800
IL-6 (pg/L)	14.4± 9.4	221.8 ± 121.2	**<0.001**

†Student's *t* test for unpaired data.

Abbreviations: FORT, Follicular Output Rate; FOI, Follicle-to-Oocyte Index; E2, Estradiol; P4, Progesterone.
